# Unlocking Testosterone Production by Biotransformation: Engineering a Fungal Model of *Aspergillus nidulans* Strain Deficient in Steroid 11α-Hydroxylase Activity and Expressing 17β-Hydroxysteroid Dehydrogenase Enzyme as Proof of Concept

**DOI:** 10.3390/biom14121502

**Published:** 2024-11-25

**Authors:** Lidia Ortega-de los Ríos, Luis Getino, Beatriz Galán, José Luis García, José M. Luengo, Alejandro Chamizo-Ampudia, José M. Fernández-Cañón

**Affiliations:** 1 Área de Bioquímica y Biología Molecular, Departamento de Biología Molecular, Universidad de León, 24007 León, Spain; lorter00@estudiantes.unileon.es (L.O.-d.l.R.); jm.luengo@unileon.es (J.M.L.); 2 Área de Genética, Departamento de Biología Molecular, Universidad de León, 24007 León, Spain; luis.getino@unileon.es; 3 Centro de Investigaciones Biológicas, Consejo Superior de Investigaciones Científicas, 28040 Madrid, Spain; bgalan@cib.csic.es (B.G.); jlgarcia@cib.csic.es (J.L.G.); 4 Institute of Molecular Biology, Genomics and Proteomics (INBIOMIC), Universidad de León, Campus de Vegazana, 24071 León, Spain

**Keywords:** *Aspergillus nidulans*, steroid 11α-hydroxylase, *Aspergillus ochraceus*, testosterone, 17β-hydroxysteroid dehydrogenase (17β-HSD) EC 1.1.1.51, androstenedione, progesterone

## Abstract

Testosterone holds significant medical and economic importance, with the global market for testosterone replacement therapies valued at approximately USD 1.9 billion in 2023. This hormone is essential for the development and maintenance of male sexual characteristics as well as bone and muscle health. It plays a key role in conditions such as hypogonadism, muscle disorders, and andropause. However, the industrial production of testosterone often involves complex chemical processes that result in low yields, high costs, and environmental damage. Microbial biotransformation of steroids presents an eco-friendly alternative to traditional chemical synthesis. A knockout strain of *Aspergillus nidulans* deficient in steroid 11α-hydroxylase activity was developed, rendering it incapable of hydroxylating androstenedione, progesterone, and testosterone. In these strains, two newly identified CYP450 enzymes, CYP68L1 from *A. nidulans* and CYP68L8 from *Aspergillus ochraceus,* were expressed to confirm their roles as steroid 11α-hydroxylases of androstenedione, progesterone, and testosterone. The availability of these 11α-hydroxylases represents significant progress toward achieving efficient single-step steroid fermentation. Furthermore, the *A. nidulans* knockout strain serves as an effective model for studying the conversion of androstenedione to testosterone upon the expression of the enzyme 17β-hydroxysteroid dehydrogenase, due to its inability to hydroxylate testosterone.

## 1. Introduction

Steroids are chemical compounds derived from cyclopentaneperhydrophenanthrene that are widely used in medicine as anti-inflammatory agents, contraceptives, anabolic steroids, hormone therapies, and immunosuppressive drugs [1,2]. Their critical role in the treatment of autoimmune diseases and specific types of cancer has been well-established [3]. The steroid market ranks second only to antibiotics in pharmaceutical importance [4]. Testosterone (TS) is considered one of the most medically significant steroids [5]. In 2023, the global market for TS therapy was valued at approximately USD 1.9 billion, with projections indicating growth to USD 2.5 billion by 2030 [6]. Current industrial methods for TS production involve multiple steps of chemical synthesis, characterized by low yields due to stereoisomerism and the formation of byproducts [7]. These methods are also more costly than biological synthesis approaches [8]. Consequently, microbial biotransformation has been employed for several years to produce novel or modified steroids, with fungal CYP450 enzymes playing a key role in these transformations [9].

The synthesis of steroids often begins with the biotransformation of phytosterols, plant-derived compounds [10], into molecules such as progesterone (PG), which undergoes partial removal of the C17 side chain, or androstenedione (AD), where the C17 side chain is entirely removed. These transformations are generally facilitated by Mycobacterium spp. and other microorganisms [4,11]. Further chemical modifications are typically required to synthesize commercially relevant compounds. One of the most critical modifications is the introduction of a hydroxyl group at the C-11 position of the sterane nucleus, which confers anti-inflammatory properties to the molecule. Steroid 11α-hydroxylation can be achieved through chemical synthesis, a method that is time-intensive and associated with higher environmental and economic costs, or through biotransformation performed by certain species of *Aspergillus* and *Rhizopus* [12,13,14]. However, the need for a second fermentation step to hydroxylate primary compounds, obtained in the first fermentation after side-chain removal, necessitates an intermediate purification, the addition of extra compounds and nutrients, and increased time and costs to achieve the final product. Therefore, the cloning of the gene encoding the steroid 11α-hydroxylase enzyme has been deemed advantageous for enabling a single-step fermentation process.

The steroid 11α-hydroxylase enzyme was cloned from *Rhizopus* (CYP509c12) several years ago [14]. This enzyme is capable of hydroxylating multiple sites within the PG steroid molecule. However, the presence of multiple hydroxylation sites complicates its industrial application due to the challenges of product separation. A similar issue has been observed with other enzymes identified in *Aspergillus ochraceus*, including CYP68AQ1 (now renamed CYP68J5), which is patented [15,16]. The CYP68J5 protein is recognized for its steroid 11α-hydroxylase activity; however, recent studies have demonstrated that when overexpressed 14-fold in *Saccharomyces cerevisiae* compared to its native expression levels in *A. ochraceus*, CYP68J5 also exhibits 7α-hydroxylase and 7,11α-bihydroxylase activities [17].

**Figure 1:**
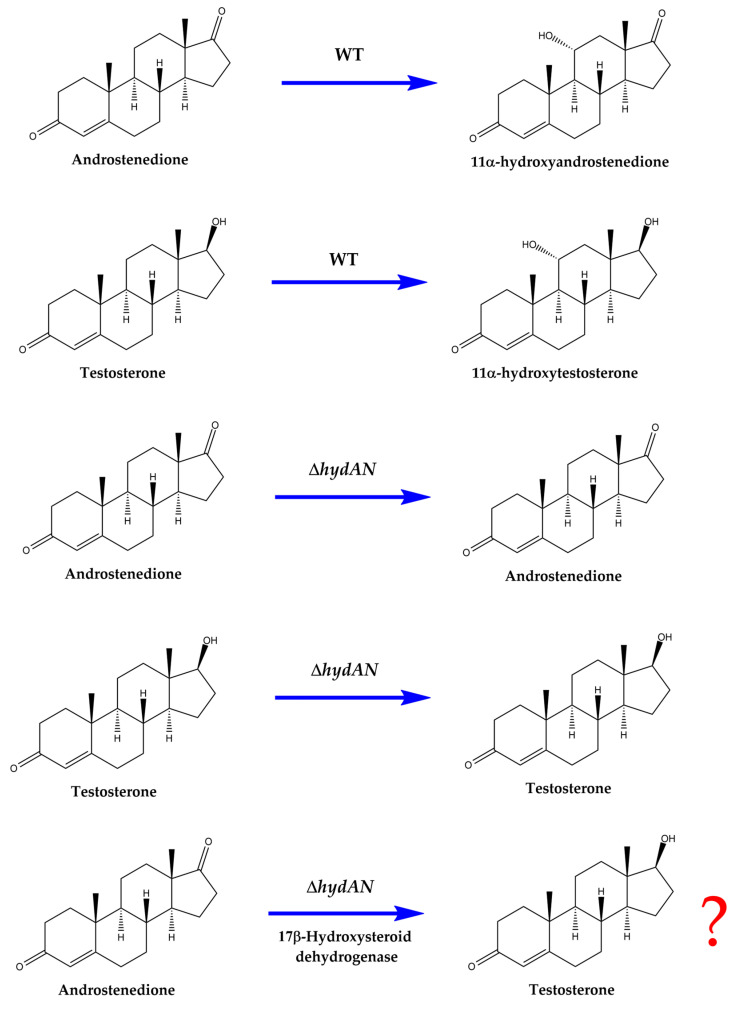
A schematic representation of reactions catalyzed by different strains of *Aspergillus nidulans*. Reactions with AD and TS are represented as mediated by the wild-type strain of *A. nidulans*, the steroid 11α-hydroxylase (CYP68L1) knockout strain of *A. nidulans* KO strain, (Δ*hydAN*), and the *A. nidulans* KO strain (Δ*hydAN*) expressing the 17β-hydroxysteroid dehydrogenase enzyme from *C. lunatus*. In the wild-type strain of *A. nidulans*, conversion occurs, whereas no conversion of AD or TS is observed in the *A. nidulans* knockout strain (Δ*hydAN*). The expression of the 17β-hydroxysteroid dehydrogenase enzyme from *C. lunatus* in the *A. nidulans* KO strain (Δ*hydAN*) could facilitate the conversion of AD to TS, as no hydroxylation of either AD or TS is produced.

Previously, we reported the cloning of steroid 11α-hydroxylase from *Aspergillus nidulans* (CYP68L1) [18]. In that study, an *A. nidulans* knockout strain lacking 11α-hydroxylase activity (KOhydAN) was developed, enabling definitive functional testing. The KOhydAN strain was unable to hydroxylate AD, PG, or TS [18] (Figure 1). It was also observed that the previously described steroid 11α-hydroxylase (CYP68J5) was unable to hydroxylate these steroids.

In this study, a novel gene encoding a steroid 11α-hydroxylase enzyme from *A. ochraceus* (CYP68L8), an industrial producer of 11α-hydroxylated steroids, is reported. This gene was expressed in the KOhydAN strain of *A. nidulans* (deficient in CYP68L1), and hydroxylation activity was restored when AD, PG, and TS were used as substrates.

The inability of the KOhydAN strain to hydroxylate TS suggests that TS can be produced without the introduction of hydroxylation into the TS molecule (i.e., without forming 11-hydroxytestosterone). For the biotransformation of AD to TS, specific enzymes capable of this conversion have been reported. One study described the expression of 17β-hydroxysteroid dehydrogenase from *Cochliobolus lunatus* (17β-hydroxysteroid: NADP 17-oxidoreductase, EC 1.1.1.51) in *M. smegmatis* [19]. Furthermore, this enzyme was expressed in the CYP68L1 knockout strain of *A. nidulans*, which lacks the ability to hydroxylate both TS and AD, allowing the evaluation of TS production from AD in this strain (Figure 1).

## 2. Materials and Methods

### 2.1. Chemical and Biochemical Reagents

The steroid compounds were either purchased from Sigma-Aldrich (San Louis, MO, USA) or received as gifts from Gadea Biopharma (León, Spain). Thermo Fisher Scientific (Wilmington, DE, USA), VWR (Radnor, PA, USA), and Condalab (Madrid, Spain) supplied all chemical reagents used for media preparation. Thermo Fisher Scientific (Wilmington, DE, USA) provided HPLC-grade acetonitrile. Molecular biology reagents were obtained from BioTools (Woburn, MA, USA) and Thermo Fisher Scientific (Wilmington, DE, USA). Macrogen (Seoul, Korea) supplied oligonucleotides.

### 2.2. Culture Conditions

To obtain spores, the fungal strains (Table 1) were cultivated on plates containing a complex medium (malt extract agar) composed of (grams per liter) 1.75 g of malt extract, 2.75 g of dextrins, 2.35 g of glycerol, 0.78 g of peptone, and 15 g of agar. Media were obtained from Pronadisa, Spain. Spores were spread on the surface of the plates and incubated at the required temperature. The spores were harvested by scraping and resuspended in 0.1% (*v*/*v*) Tween 80. They were then washed in 0.1% (*v*/*v*) Tween 80 and collected by centrifugation (3500× *g*) before being resuspended in 0.1% (*v*/*v*) Tween 80. A chemically defined medium (MM) consisting of KH_2_PO_4_ 1.36 g/L, (NH_4_)_2_SO_4_ 2 g/L, MgSO_4_·7H_2_O 0.25 g/L, and Hunter salts 1 mL/L was used [18,20]. For fermentations, the potassium phosphate concentration was increased to 100 mM (13.6 g/L) to prevent pH fluctuations during fermentation. For the preparation of solid media, 1.5% (*w*/*v*) agar was added. *A. nidulans* was cultured at 37 °C, while *A. ochraceus* and *C. lunatus* were grown at 32 °C [20]. All liquid fermentation media were inoculated with 10⁶ spores/mL.

### 2.3. DNA Manipulation

All DNA manipulations were performed using standard protocols or commercial kits. Plasmid DNA was isolated using the NucleoSpin Plasmid Extract kit (Macherey-Nagel, Dueren, Germany). According to the manufacturer’s instructions, DNA fragments from agarose gel and PCR reactions were purified using the NucleoSpin Gel and PCR Clean-Up kit (Macherey-Nagel, Dueren, Germany). *Escherichia coli* XL-1 Blue was used as the host for plasmid propagation and molecular biology manipulations. LB medium (Thermo Fisher Scientific, Wilmington, DE, USA), supplemented with the appropriate antibiotics, was used, and cultures were incubated at 37 °C. *E. coli* cells were transformed by electroporation, following the protocol described by Miller and Nickoloff [21]. Recombinant strains of *A. nidulans* were generated according to established protocols [18,22] (Table 1).

RNA extraction was carried out using TRIzol reagent (Invitrogen, Wilmington, DE, USA) according to the manufacturer’s instructions. First-strand cDNA was synthesized using the First Strand cDNA Synthesis Kit (Thermo Fisher Scientific, Wilmington, DE, USA) from extracted RNA with oligo dT primers. This cDNA was then used to amplify the target gene with specific primers (Table 2). PCR conditions were optimized according to the primers used. These primers included restriction site sequences (*NcoI* and *EcoRI)* to facilitate gene cloning into the expression plasmid (p1660) (Table 3). The glyceraldehyde phosphate dehydrogenase A (*gpdA*) promoter from *A. nidulans* was contained in the p1660 plasmid to drive the expression of cloned genes, and the *pyroA* gene was used as the selection marker. The p1660 plasmid was provided by Dr. M. A. Peñalva (Centro de Investigaciones Biológicas, CSIC, Madrid, Spain) [23]. PCR amplifications were routinely performed using Phire Polymerase and Phusion Polymerase (Thermo Fisher Scientific, Wilmington, DE, USA). The Maxima H Minus First Strand cDNA Synthesis Kit with DNase (Thermo Fisher Scientific, Wilmington, DE, USA) was employed to generate double-stranded cDNA for subtractive hybridization [18]. When necessary, mRNA was isolated using the Mag-Bind mRNA Kit (VWR Omega, Radnor, PA, USA) with magnetic bead enrichment.

### 2.4. HPLC Analysis

Culture broth analysis was performed as described by Ortega-De los Ríos et al. (2017) [18]. Samples of culture medium (5 mL) were mixed in a 1:1 ratio with ethyl acetate. The organic phase was then isolated and evaporated, and the resulting pellet was dissolved in an equivalent volume of ethanol. Samples were filtered through a 0.22 μm PTFE syringe filter before the HPLC analysis [18].

HPLC analyses were conducted using an Alliance 2690 HPLC system coupled to a 996 photodiode array detector (Waters). A Kromasil 100 C18 5 µm 25 × 0.46 cm column, along with a Nucleosil C18 5 µm 1 × 0.46 cm precolumn, was employed. Isocratic flow (1.5 mL/min) of acetonitrile and water (60:40 by volume) was used, and data processing was carried out using Empower3 software (Waters) [18]. Under these conditions, the retention times were as follows (in minutes): AD, 5.3 ± 0.3; 11α-hydroxylation of AD (11-OHAD), 2.6 ± 0.2; PG, 10.8 ± 0.5; 11α-hydroxylation of PG (11-OHPG), 3.4 ± 0.3; and TS, 4.3 ± 0.2.

For the quantification of AD and TS, standard straight lines were performed. For each concentration (0.1, 0.25, 0.5, 0.75, 1, 1.5, and 2 mg/mL) triplicates dissolved in ethanol were performed. Under the same chromatographic conditions, each sample was analyzed in duplicate. The R^2^ value for AD and TS was 0.99 in both cases, while the equations of the lines were AU = 56,710,328.92X + 3,839,386.23 and AU = 9,700,119.63X + 1,356,852.62, respectively. For TS quantification, measurements were taken directly from the extraction, whereas for AD, measurements were taken directly and from a 1/5 dilution to ensure that at least one of the values fell within the calibration curve range.

### 2.5. Bioinformatic Analysis

BLAST software (BLAST+ 2.16.0) [24] and the NCBI Learn page (www.ncbi.nlm.nih.gov/learn/ (accessed on 25 September 2024)) were used to search for homologous genes and proteins among the microorganisms studied. The selected sequences were aligned using BioEdit software version 7.2.6.1 (Carlsbad, CA, USA) with native ClustalW (version 1.4, Heidelberg, Germany) [25]. The results and their graphical representations were analyzed using GraphPad Prism v.6 software (GraphPad Software, Inc., Boston, MA, USA).

## 3. Results

### 3.1. Aspergillus nidulans Exhibits an Inducible Steroid 11α-Hydroxylase Activity That Is Not Replaced by CYP68J5 from Aspergillus ochraceus

The 11α-hydroxylation of steroids has been described in several species within the *Aspergillus* genus. Fermentation media from *A. nidulans* and *A. ochraceus* supplemented with AD, PG, and TS were previously analyzed using HPLC, confirming the presence of 11α-hydroxylated derivatives of AD, PG, and TS [18].

**Figure 2:**
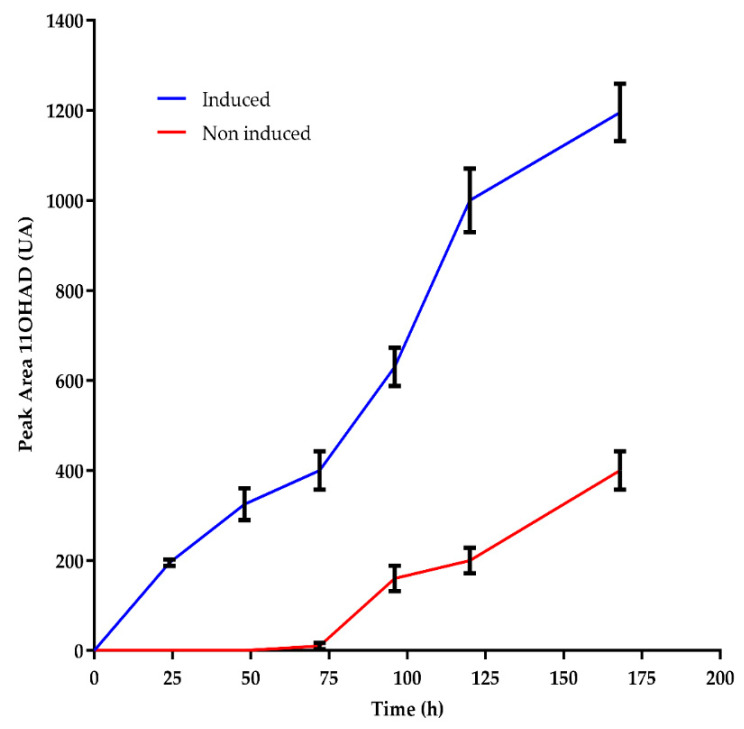
Appearance of 11α-hydroxylated androstenedione from AD in *A. nidulans* cultures previously grown with AD (blue) and without AD (red), and then transferred to media with AD. The number of biological replicates was n = 3. HPLC measurements were taken at a wavelength of 240 nm.

When *A. nidulans* cultures grown in minimal media with or without 1 g/L of AD were transferred to media containing AD, a delay in the onset of steroid hydroxylase activity was observed in cultures previously grown without AD. This finding suggests that the induction of specific enzymes is required for AD hydroxylation (Figure 2). Nearly undetectable levels of mRNA expression of the *hydAN* (CYP68L1, Table 2) gene were observed in non-induced cultures grown in the absence of AD, TS, and PG. In induced cultures, the appearance of mRNA was used to clone the *hydAN* gene through suppression subtractive hybridization [18].

**Figure 3:**
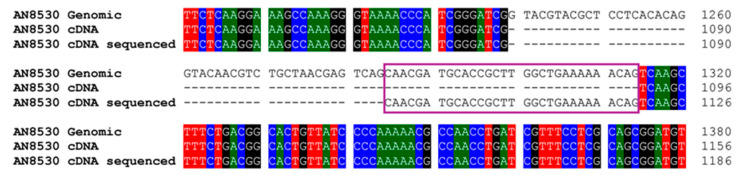
Alignment of genomic DNA, cDNA obtained from database, and cDNA sequence obtained by us from AN8530 (CYP68L1) from *A. nidulans* in this study. The portion of the exon expansion is shown in this figure.

The cDNA sequence of the *A. nidulans hydAN* gene, responsible for the 11α-hydroxylation of TS, AD, and PG by a CYP450 enzyme designated as CYP68L1, revealed discrepancies when compared to the predicted intron structure in the database. Specifically, one intron was shorter than expected, and the subsequent exon began 30 nucleotides earlier than predicted (Figure 3 and Figure S1). To confirm that this gene encoded the steroid 11α-hydroxylase enzyme, a knockout strain lacking this gene (KOhydAN; Table 1) was previously obtained. KOhydAN strain showed no hydroxylase activity against AD, TS, or PG [18]. Dr. David Nelson (Department of Biochemistry, University of Tennessee, Memphis, USA) officially designated this CYP450 (encoded by *hydAN* from *A. nidulans*) as CYP68L1 (GenBank MF153379) [26].

**Figure 4:**
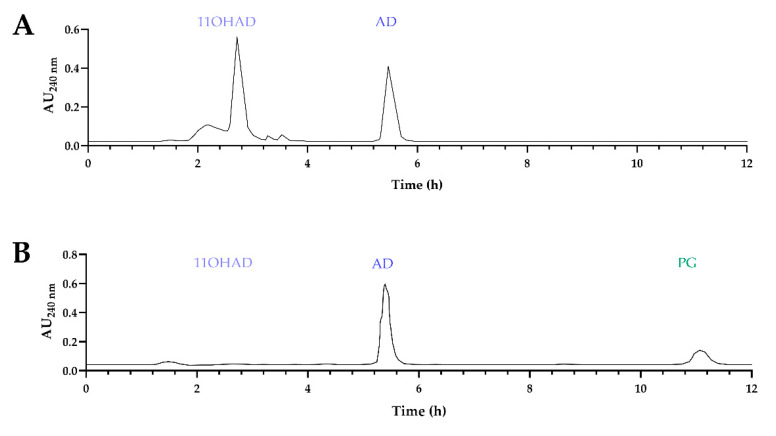

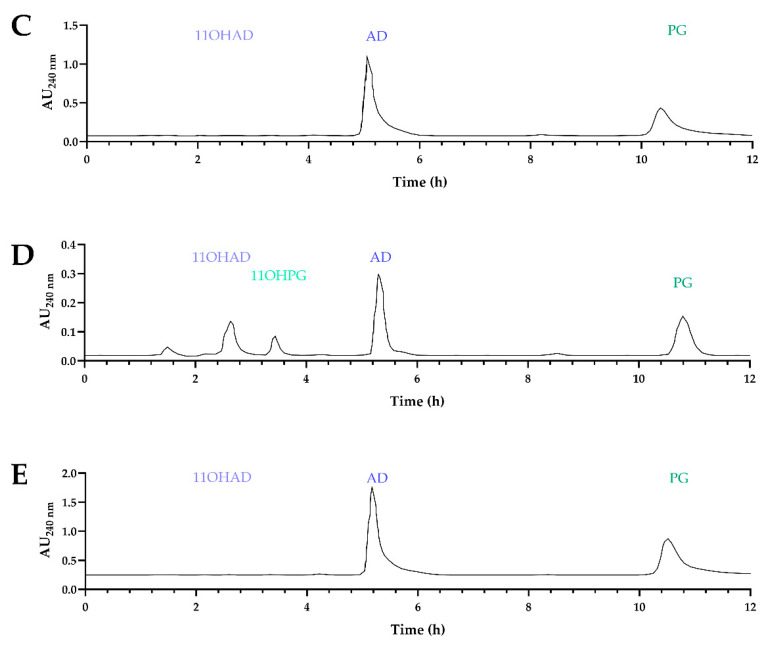
Chromatograms obtained from the analysis of fermentation media of strains (72 h): (**A**) KOhydAN *+* hydAN at 32 °C, (**B**) KOhydAN *+* hydJ5AO at 32 °C and (**C**) at 37 °C, (**D**) KOhydAN +hydAO *at* 32 °C, and (**E**) KOhydAN + hydAO *at* 37 °C. The chromatograms of the culture media containing AD (blue), PG (green), and the KOhydAN expressing the CYP68L8 and CYP68J5 proteins (from *A. ochraceus*) were analyzed. The construction of the KOhydAN expressing CYP68L1 protein was analyzed in media with AD to verify the recovery of activity. New peaks were identified in the chromatogram results, including the 11α-hydroxylation of AD (11-OHAD) in light blue and the 11α-hydroxylation of PG (11-OHPG) in light green. Temperatures of 32 °C and 37 °C were tested to determine the optimal enzymatic activity of *A. ochraceus* proteins expressed in *A. nidulans*, reflecting the different growth temperatures of these *Aspergillus* species.

Hydroxylase activity was restored by expressing the *hydAN* gene encoding this CYP450 in the KOhydAN strain (KOhydAN + hydAN, Table 1) (Figure 4A). Additionally, a previously described and patented protein with steroid 11α-hydroxylase activity from *A. ochraceus* was identified [15]. This patented protein, CYP68J5 (*hydJ5AO*, Table 2), was expressed in the *A. nidulans* KOhydAN strain (KOhydAN + hydJ5AO, Table 1), and fermentations were conducted at 32 °C and 37 °C, the optimal growth temperatures for *A. ochraceus* and *A. nidulans*, respectively. No optimal temperature for the enzymatic activity of these proteins was available. This approach allowed for the evaluation of which temperature was more suitable for expressing proteins from *A. ochraceus* in *A. nidulans* (Figure 4B,C). It was concluded that this protein (CYP68J5) did not restore hydroxylation capability at the 11α position of steroid molecules (AD and PG) under the conditions tested. However, the emergence of a peak at an early retention time (1.5 min) was observed at 32 °C, which could be attributed to other types of hydroxylation. 

### 3.2. Identification of a New CYP450 with Steroid 11α-Hydroxylase Activity from Aspergillus ochraceus

**Figure 5:**
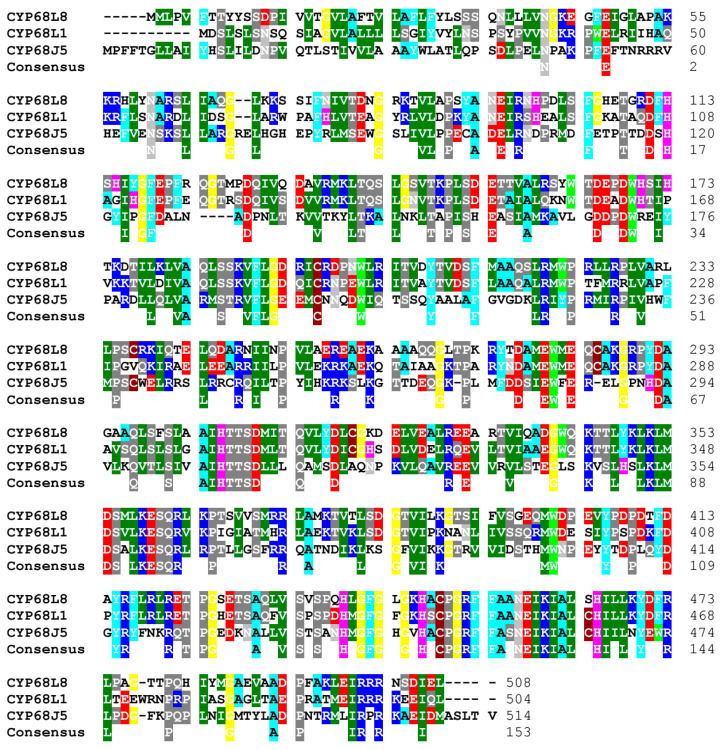
Alignment of proteins: CYP68L1 from *A. nidulans* with CYP68J5 (CYP68AQ1) and CYP68L8 from *A. ochraceus*. The identity of CYP68L1 with CYP68J5 was 36%. CYP68L1’s identity with CYP68L8 was 62%.

As demonstrated in previous studies, *Aspergillus ochraceus* exhibits steroid 11α-hydroxylase activity [18]; however, this activity does not appear to be associated with the patented CYP450 enzyme (CYP68J5). This prompted an investigation into the genome of *A. ochraceus* to identify the ortholog of *Aspergillus nidulans* CYP450L1 hydroxylase. A CYP450 with a high identity (62%) to *A. nidulans* CYP68L1 was identified, isolated, and named *hydAO* (Table 2). The complete *hydAO* gene was sequenced. Dr. Nelson (Department of Biochemistry, University of Tennessee, Memphis, USA) designated this enzyme as CYP68L8 (GenBank MF153380). In contrast, the putative steroid 7,11α-bihydroxylase (the patented CYP450, CYP68J5) exhibited a low identity (36%) as compared to CYP68L8 protein (encoded by *hydAO* gene) (Figure 5). 

After identifying the *hydAO* (CYP68L8) sequence from *Aspergillus ochraceus*, it was expressed in the KOhydAN strain of *A. nidulans* under the same conditions as the previously obtained strains, resulting in the ΔhydAN + hydAO strain (Table 1). Fermentations of KOhydAN + hydAO were conducted at 32 °C and 37 °C, following the same rationale applied to the patented CYP68J5 protein in the ΔhydAN + hydJ5AO strain. This decision was based on the lack of evidence regarding the optimal temperature for the enzymatic activity of these *A. ochraceus*-derived proteins expressed in *A. nidulans*. This approach enabled the assessment of which temperature was more suitable for the expression of proteins originating from *Aspergillus ochraceus* and *Aspergillus nidulans*. Chromatograms of the fermentation products indicated that hydroxylase activity was restored for CYP68L8 at the optimal temperature of 32 °C (Figure 4D,E).

### 3.3. Production of Testosterone from the Steroid 11α-Hydroxylase Knockout Strain of A. nidulans Expressing the Enzyme 17β-Hydroxysteroid-Dehydrogenase (EC 1.1.1.51) from Cochliobolus lunatus (Curvularia lunata)

Fernández-Cabezón et al. [19] demonstrated the production of testosterone (TS) from androstadienedione (AD) by *Mycobacterium smegmatis* expressing the 17β-hydroxysteroid dehydrogenase from *Cochliobolus lunatus*. For this reason, the *hsdCL* gene of the 17β-hydroxysteroid dehydrogenase (EC 1.1.1.51) from *Cochliobolus lunatus* and the *hsdAN* gene of the putative 17β-hydroxysteroid dehydrogenase from *Aspergillus nidulans* were expressed in the KOhydAN to assess the feasibility of obtaining pure TS from AD (Table 2). The search for this putative 17β-hydroxysteroid dehydrogenase from *Aspergillus nidulans* was based on previous studies with *A. nidulans* (WTAn, Table 1), where WTAn was shown to convert TS into AD through the action of 17β-hydroxysteroid dehydrogenase enzymes. These enzymes are capable of catalyzing the conversion of AD to TS or TS to AD [18].

To identify the endogenous 17β-hydroxysteroid dehydrogenase in *Aspergillus nidulans*, homology searches (by BLAST) were performed with other 17β-hydroxysteroid dehydrogenases documented in the literature. One of these was the 17β-hydroxysteroid dehydrogenase from *Cochliobolus lunatus* (*hsdCL*, GenBank accession: AAD12052) (Table 2), which exhibited 40% amino acid identity with a gene in *A. nidulans* (*hsdAN*, GenBank accession: CAK469791). This *hsdAN* gen showed 32% identity with the 17β-hydroxysteroid dehydrogenase from *Comamonas testosteroni* (17β-HSDCt, GenBank accession: 1HXHA) and 37% identity with the 17β-hydroxysteroid dehydrogenase from *Aspergillus niger* (17β-HSD of A. niger, GenBank accession: CAK46979.1).

**Figure 6:**
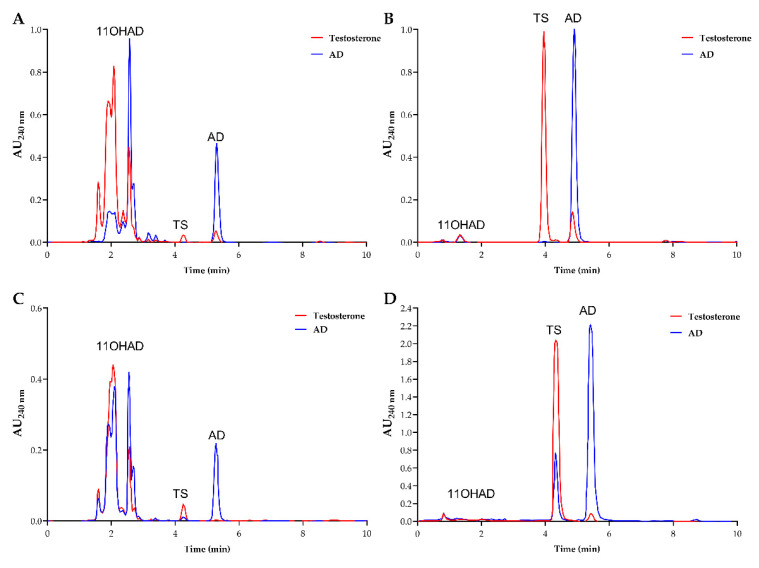
Chromatograms obtained from the analysis of fermentation media (72 h) of (**A**) WTAn + hsdAN; (**B**) KOhydAN + hsdAN; (**C**) WTAn + hsdCL; and (**D**) KOhydAN + hsdCL supplemented with AD (blue) and added TS (red).

When TS was added to the media with wild-type *A. nidulans* strain expressing 17β-HSD from *A. nidulans* (WTAn *+* hsdAN, Table 1), several compounds were identified in the fermentation medium, including 11α-hydroxylated testosterone (11OHTS) [18]. An analysis of these fermentation media indicated the production of hydroxylated compounds from AD and from TS (Figure 6A). However, no modifications of TS were detected in the culture medium of the KOhydAN + hsdAN strain (Table 1, Figure 6B), with only a small amount of AD observed, likely due to the reversible 17β-hydroxysteroid dehydrogenase activity of *A. nidulans*, which generates AD from TS. When the WTAn + hsdCL strain (expressing 17β-hydroxysteroid dehydrogenase from *C. lunatus,* Table 1) was used in the fermentation, hydroxylated compounds were also produced (Figure 6C). However, when the KOhydAN + hsdCL strain was used (expressing 17β-HSD from *C. lunatus*, Table 1) no hydroxylated compounds were detected in the fermentation media, and this KOhydAN + hsdCL strain was capable of converting AD to TS with high efficiency (Figure 6D).

**Figure 7:**
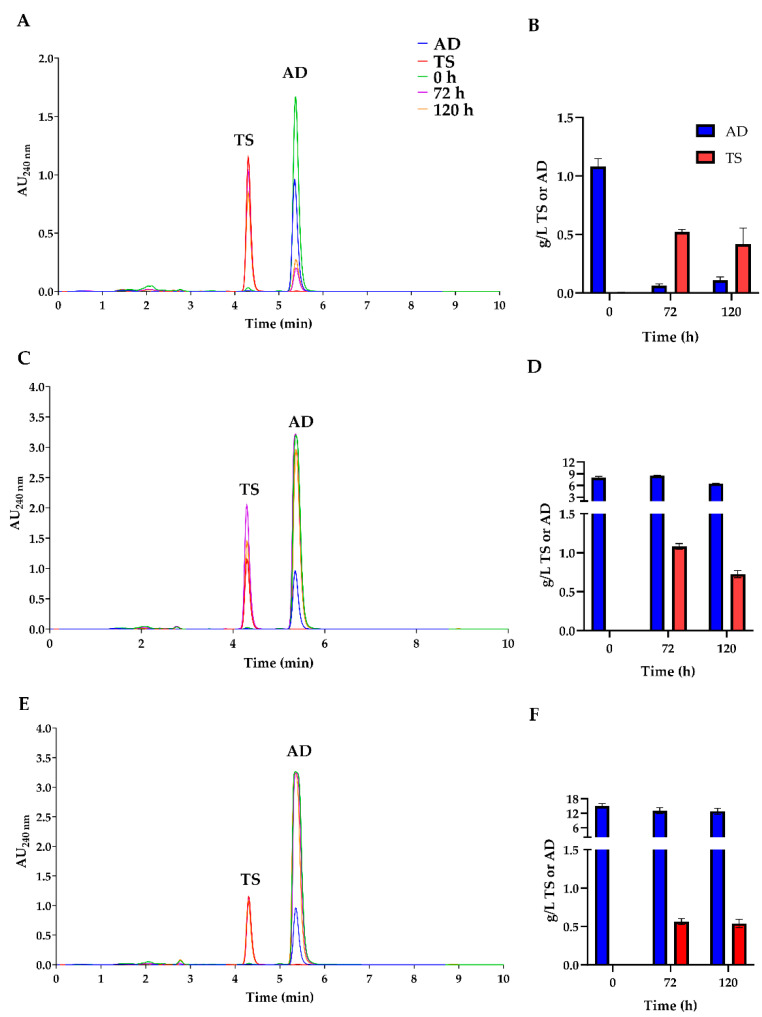
Chromatograms obtained from the analysis of fermentation media of KOhydAN + hsdCL. The transformation of AD to TS was measured at 0 h (green), 72 h (purple), and 120 h (orange). AD (blue) and TS (red) standards are shown. Different initial concentrations of AD added to the media were added: (**A**) 1 g/L of AD, (**C**) 8 g/L of AD, and (**E**) 15 g/L of AD. The TS production in g/L was calculated for each initial AD concentration: (**B**) 1 g/L of AD, (**D**) 8 g/L of AD, and (**F**) 15 g/L of AD.

Additionally, fed fermentations with media supplemented with 0.1% AD converted more than 54% of AD into TS, a more valuable compound (Figure 7B). However, increasing the amount of AD led to a decrease in TS production, likely due to toxic effects or growth retardation caused by high concentrations of AD in the fermentation medium (Figure 7). At higher concentrations, AD exhibits low solubility, which hinders the ability to obtain a representative sample of the total AD present in the broth for HPLC measurements [27].

## 4. Discussion

This study underscores the possibility to produce TS by biotransformation by using, as a model, a manipulated strain from *A. nidulans*, with two major modifications. Additionally, we also defined distinct roles of two enzymes steroid 11α-hydroxylases, CYP68L1 from *Aspergillus nidulans* and CYP68J5 from *Aspergillus ochraceus* in the hydroxylation of some steroid molecules, specifically AD, TS, and PG [16,17,28,29]. The analysis of the fermentation media in *A. nidulans* supplemented with AD and PG confirmed the production of 11α-hydroxylated derivatives of AD and PG catalyzed by CYP68L1 [1,18]. Hydroxylation activity in *A. nidulans* was rapidly induced by steroids such as AD (Figure 2), although a delayed onset was observed in cultures transferred to AD-containing media. This observation suggests that enzyme induction is essential for hydroxylation [2]. The successful cloning of the steroid 11α-hydroxylase gene using SSH highlights the importance of inducible enzymes in identifying relevant CYP450 genes [18,30,31]. Minor discrepancies between the cloned CYP450 cDNA and its predicted intron structure, aligned with the CYP68L1 sequence of *A. ochraceus*, were resolved in this study (Figure 3). The creation of a CYP68L1 knockout strain, followed by the restoration of its activity, confirmed the enzyme’s central role in the hydroxylation of AD, TS, and PG [18] (Figure 4A). In contrast, the expression of CYP68J5 in the KOhydAN strain did not restore steroid 11α-hydroxylase activity against these steroids, as demonstrated in Figure 4B,C.

Reports indicate that the overexpression of CYP68J5 in *A. ochraceus* enables the hydroxylation of steroids such as D-ethylgonendione and 16,17α-epoxyprogesterone [28,29], and that recombinant *Saccharomyces cerevisiae* strains expressing CYP68J5 similarly achieve the hydroxylation of these substrates [16]. However, the expression of *hydJ5AO* in the KOhydAN strain (KOhydAN + hydJ5AO) did not yield detectable 11α-hydroxylase activity (Figure 4B,C). Previous studies suggest that only high levels of CYP68J5 expression up to 14 times those found in wild-type *A. ochraceus* result in AD 11α-, 7α-, or 11,7α-hydroxylation [15,17]. Thus, while CYP68J5 exhibits some hydroxylation capacity, it does not achieve CYP68L1’s activity levels with AD and PG and has broader substrate specificity, potentially functioning as a mono- or dihydroxylase [17,28,29].

The identification of CYP68L8 in *A. ochraceus*, closely related to CYP68L1 (Figure 5), supports the existence of diverse hydroxylase activities within the *Aspergillus* genus (Figure 4D,E). The ability of CYP68L8 to restore steroid 11α-hydroxylase activity in the *A. nidulans* knockout strain (KOhydAN) underscores its potential for industrial applications in steroid biotransformation [4,9]. *A. nidulans* is widely regarded as a model organism for metabolic and genetic studies, with its mutant library facilitating steroid biosynthesis research. Studies with *A. nidulans* have provided insights into human diseases such as tyrosinemia and alkaptonuria [31,32], as well as the penicillin biosynthetic pathway [33]. The capacity of *Aspergillus* strains to introduce hydroxyl groups at specific positions on steroid molecules highlights their significance in the industrial production of steroids [13].

The cloning of the 11α-steroid hydroxylase gene represents a step forward in developing an environmentally friendly system capable of efficient steroid transformations. Previous attempts to clone this enzyme were unsuccessful, leaving whole-cell biocatalysis the preferred method for steroid hydroxylation [34]. The identification of the 11α-hydroxylase gene advances the goal of achieving single-step steroid fermentation.

The characterization of the 11α-hydroxylase-deficient KOhydAN strain revealed that TS was not hydroxylated [18]. This 11α-hydroxylase mutant allowed the KOhydAN + hsdCL strain to efficiently convert AD to TS through the expression of 17β-hydroxysteroid dehydrogenase from *C. lunatus*, resulting in the production of a valuable product without hydroxylated byproducts (Figure 6D). However, higher AD concentrations resulted in decreased TS production, likely due to toxic effects on *A. nidulans* (Figure 7). This finding suggests that the KOhydAN strain is a useful model for studying TS production via biotransformation. However, future research must explore the use of *A. ochraceus*, which exhibits greater tolerance to high steroid concentrations, to improve production efficiency [4,28].

## 5. Conclusions

Steroid production through microbial biotransformation has been established as a promising alternative to traditional chemical methods due to its reduced environmental impact, specificity, and enhanced efficiency. In this study, two cytochrome P450 (CYP450) enzymes with steroid 11α-hydroxylase activity were cloned and characterized: CYP68L1 from *Aspergillus nidulans* and CYP68L8 from *Aspergillus ochraceus*. Both enzymes were found to effectively hydroxylate steroids, including androstadienedione (AD) and progesterone (PG). Although the patented CYP450 enzyme from *A. ochraceus*, CYP68J5, did not demonstrate 11α-hydroxylating activity on these steroids, the characterization of CYP68L8 represents a significant step forward in advancing single-step fermentation processes for the production of hydroxylated steroids.

Additionally, a mutant strain of *A. nidulans*, which lacks steroid 11α-hydroxylase activity but expresses the 17β-hydroxysteroid dehydrogenase from *C. lunatus*, was demonstrated to efficiently convert AD into testosterone (TS), thereby opening new avenues for industrial TS production. These results suggest that the biosynthesis of TS can be utilized for therapeutic and commercial applications, which could reduce costs and enhance production yields.

However, despite the value of TS as a steroid compound used in various therapeutic applications, *A. nidulans* is not considered an ideal industrial producer due to its sensitivity to high steroid concentrations in the medium. In contrast, *A. ochraceus* has been recognized as an efficient industrial producer of steroids, which has prompted the cloning of its steroid 11α-hydroxylase gene. This enzyme exhibits similar characteristics and substrate specificity to that of *A. nidulans*, further establishing *A. ochraceus* as a more viable option for large-scale steroid production through biotransformation.

## Figures and Tables

**Table 1:** Strains used in this study.

Strain	Description	Reference
WTAn	Δ*nkuA*, *pyrG89, pyroA4* (*Aspergillus nidulans*)	[18]
KOhydAN	Δ*nkuA*, *pyroA4,* Δ*hydAN* (*Aspergillus nidulans*)	[18]
WTAo	Wild-type strain (*Aspergillus ochraceus*)	Colección Española de Cultivos Tipo (ATCC 1008)
WTCl	Wild-type strain (*Cochliobolus lunatus*)	Colección Española de Cultivos Tipo (M 118)
KOhydAN + hydAN	Δ*nkuA*, Δ*hydAN,* p1660-*gpdA* promoter::*hydAN* **(KO strain** (Δ*hydAN*) **expressing CYP68L1)**	This work.
KOhydAN + hydAO	Δ*nkuA*, Δ*hydAN,* p1660*-gpdA* promoter::*hydAO* **(KO strain** (Δ*hydAN*) **expressing CYP68L8)**	This work.
KOhydAN + hydJ5AO	Δ*nkuA*, Δ*hydAN,* p1660*-gpdA* promoter::CYP68J5 **(KO strain** (Δ*hydAN*) **expressing CYP68J5)**	This work.
WTAn + hsdAN	Δ*nkuA*, *pyrG89,* p1660*-gpdA* promoter::17β-HSD **(WTAn expressing 17-βHSDAn)**	This work
KOhydAN + hsdAN	Δ*nkuA*, Δ*hydAN,* p1660*-gpdA* promoter::17β-HSDAn **(KO strain** (Δ*hydAN*) **expressing 17β-HSDAn)**	This work.
WTAn + hsdCL	Δ*nkuA*, *pyrG89,* p1660*-gpdA* promoter::17β-HSDCl **(WTAn expressing 17-βHSDCl)**	This work
KOhydAN + hsdCL	Δ*nkuA*, Δ*hydAN,* p1660*-gpdA* promoter::17β-HSD **(KO strain** (Δ*hydAN*) **expressing 17-βHSDCl)**	This work.

**Table 2:** Genes used in this study.

Enzyme	Gen	Protein	Accession (GenBank)	Reference
Steroid 11α-hydroxylase *A. nidulans*	*hydAN*	CYP68L1	MF153379	[18]
Steroid 11α, 7α bihydroxylase *A. ochracecus*	*hydJ5AO*	CYP68J5	DD180525	[17]
Steroid 11α-hydroxylase *A. ochraceus*	*hydAO*	CYP68L8	MF153380	This work
17β-Hydroxysteroid dehydrogenase *A. nidulans*	*hsdAN*	17β-HSDAn	XM658962	This work
17β-Hydroxysteroid dehydrogenase *C. lunatus*	*hsdCL*	17β-HSDCl	AF069518	[19]

**Table 3:** Primers (red color indicates restriction sites NcoI and EcoRI). A single-step annealing and extension PCR was conducted at a temperature of 72 °C, as recommended by the polymerase manufacturer.

Protein	Forward	Reverse
CYP68L1	5′-cattaccatggatagcctttcgttatcaaactcc-3′	5′-cgggaattcctataactgaatttcctcttttctcc-3′
CYP68L8	5′-cattaccatggtgctcccagtattcacgacg-3′	5′-cgggaattctcatagttcaatgtcggagtttctcc-3′
CYP68J5	5′-cattaccatggccttcttcactgggct-3′	5′-gcgggaattctcagcatcctggtattg-3′
17β-HSDAn	5′-gacttccatggcgctcgccggaaaa-3′	5′-gcgggaattcctatgccattcccccgt-3′
17β-HSDCl	5′-gactcccatggcgcacgtggagaacgcct-3′	5′-gcgggaattcttaggcggcgccgccgtcca-3′

## Data Availability

All relevant data are included in the manuscript.

## Supplementary Materials

The following supporting information can be downloaded at: https://www.mdpi.com/article/10.3390/biom14121502/s1, Figure S1: Alignment of genomic, database cDNA, and sequenced cDNA for CYP68L1 from *A. nidulans* in this study.
