# Supplementary material for: Unlocking Testosterone Production by Biotransformation: Engineering a Fungal Model of Aspergillus nidulans Strain Deficient in Steroid 11α-Hydroxylase Activity and Expressing 17β-Hydroxysteroid Dehydrogenase Enzyme as Proof of Concept
Source: Biomolecules. 2024 Nov 25;14(12):1502. doi: 10.3390/biom14121502 (PMC11673057; doi:10.3390/biom14121502)
Supplement: Supplementary file 1 [file biomolecules-14-01502-s001.zip › biomolecules-3257474-Supplementary Figure S1.pdf]

```
AN8530 Genomic          ATGGATAGCC TTTCGTTATC AAACTCCCAA TCTATTGCTG GCGTGCTAGC TCTGTTGCTG   60
AN8530 cDNA             ATGGATAGCC TTTCGTTATC AAACTCCCAA TCTATTGCTG GCGTGCTAGC TCTGTTGCTG   60
AN8530 cDNA sequenced   ATGGATAGCC TTTCGTTATC AAACTCCCAA TCTATTGCTG GCGTGCTAGC TCTGTTGCTG   60

AN8530 Genomic          CTGAGCGGCA TATATGTGTA CCTGAACTCG CCCTCATACC CGGTTGTCAA TGGGAAACGC   120
AN8530 cDNA             CTGAGCGGCA TATATGTGTA CCTGAACTCG CCCTCATACC CGGTTGTCAA TGGGAAACGC   120
AN8530 cDNA sequenced   CTGAGCGGCA TATATGTGTA CCTGAACTCG CCCTCATACC CGGTTGTCAA TGGGAAACGC   120

AN8530 Genomic          CCATGGGAGC TCCGAATAAT CCATGCGCAG AAGCGGTTCC TATCGAATGC GCGCGATCTA   180
AN8530 cDNA             CCATGGGAGC TCCGAATAAT CCATGCGCAG AAGCGGTTCC TATCGAATGC GCGCGATCTA   180
AN8530 cDNA sequenced   CCATGGGAGC TCCGAATAAT CCATGCGCAG AAGCGGTTCC TATCGAATGC GCGCGATCTA   180

AN8530 Genomic          ATCGATTCGG GTCTTGCGAG GGTAAGAGTT CCCATTGAAT ACATCCATAG GTTTGAGCTG   240
AN8530 cDNA             ATCGATTCGG GTCTTGCGAG G--------- ---------- ---------- ----------   201
AN8530 cDNA sequenced   ATCGATTCGG GTCTTGCGAG G--------- ---------- ---------- ----------   201

AN8530 Genomic          ACAAGTATTG CGCAAAGTGG CCAGCCTTTC ACCTTGTCAC TGAGGCAGGA TACAGACTTG   300
AN8530 cDNA             ---------- -------TGG CCAGCCTTTC ACCTTGTCAC TGAGGCAGGA TACAGACTTG   244
AN8530 cDNA sequenced   ---------- -------TGG CCAGCCTTTC ACCTTGTCAC TGAGGCAGGA TACAGACTTG   244

AN8530 Genomic          TCCTGGACCC CAAGTATGCA AACGAAATTC GGAGTCATGA GGCACTGAGC TTTGGCAAGG   360
AN8530 cDNA             TCCTGGACCC CAAGTATGCA AACGAAATTC GGAGTCATGA GGCACTGAGC TTTGGCAAGG   304
AN8530 cDNA sequenced   TCCTGGACCC CAAGTATGCA AACGAAATTC GGAGTCATGA GGCACTGAGC TTTGGCAAGG   304

AN8530 Genomic          CAACTGCACA AGACTTCCAC GCCGGCATAC ACGGATTCGA GCCCTTTGAA CAGGGAACCA   420
AN8530 cDNA             CAACTGCACA AGACTTCCAC GCCGGCATAC ACGGATTCGA GCCCTTTGAA CAGGGAACCA   364
AN8530 cDNA sequenced   CAACTGCACA AGACTTCCAC GCCGGCATAC ACGGATTCGA GCCCTTTGAA CAGGGAACCA   364

AN8530 Genomic          GGTCGGACCA GATCGTTTCC GATGTGGTTA GGATGAAGTT GACCCAGAGT CTAGGTAGGG   480
AN8530 cDNA             GGTCGGACCA GATCGTTTCC GATGTGGTTA GGATGAAGTT GACCCAGAGT CTAG------   419
AN8530 cDNA sequenced   GGTCGGACCA GATCGTTTCC GATGTGGTTA GGATGAAGTT GACCCAGAGT CTAG------   419

AN8530 Genomic          CCATTGAACC TTGGAATAAG TATTGAGCTG ACTTGGCAAG GGAATGTGAC GAAGCCACTG   540
AN8530 cDNA             ---------- ---------- ---------- ---------- GGAATGTGAC GAAGCCACTG   438
AN8530 cDNA sequenced   ---------- ---------- ---------- ---------- GGAATGTGAC GAAGCCACTG   438

AN8530 Genomic          TCTGATGAGA CTGCGATTGC TCTTCAAAAG AACTGGACAG ATGAAGCTGG TGAGTCACCA   600
AN8530 cDNA             TCTGATGAGA CTGCGATTGC TCTTCAAAAG AACTGGACAG ATGAAGCTG- ----------   487
AN8530 cDNA sequenced   TCTGATGAGA CTGCGATTGC TCTTCAAAAG AACTGGACAG ATGAAGCTG- ----------   487

AN8530 Genomic          CTCTTTATTA CGGGACGGTC TACTGACGTA ACATAGACTG GCACACAATC CCTGTCAAGA   660
AN8530 cDNA             ---------- ---------- ---------- ------ACTG GCACACAATC CCTGTCAAGA   511
AN8530 cDNA sequenced   ---------- ---------- ---------- ------ACTG GCACACAATC CCTGTCAAGA   511

AN8530 Genomic          AGACCGTCCT CGATATCGTC GCCCAGTTAT CGTCCAAGGT CTTCCTAGGT GACCAAATCT   720
AN8530 cDNA             AGACCGTCCT CGATATCGTC GCCCAGTTAT CGTCCAAGGT CTTCCTAGGT GACCAAATCT   571
AN8530 cDNA sequenced   AGACCGTCCT CGATATCGTC GCCCAGTTAT CGTCCAAGGT CTTCCTAGGT GACCAAATCT   571

AN8530 Genomic          GCCGCAACCC CGAGTGGCTC CGGATCACGG TGGCCTATAC GGTGGACTCG TTTCTAGCCG   780
AN8530 cDNA             GCCGCAACCC CGAGTGGCTC CGGATCACGG TGGCCTATAC GGTGGACTCG TTTCTAGCCG   631
AN8530 cDNA sequenced   GCCGCAACCC CGAGTGGCTC CGGATCACGG TGGCCTATAC GGTGGACTCG TTTCTAGCCG   631

AN8530 Genomic          CACAAGCATT GCGCATGTGG CCAACCTTCA TGCGACGCCT CGTCGCCCCA TTTATCCCTG   840
AN8530 cDNA             CACAAGCATT GCGCATGTGG CCAACCTTCA TGCGACGCCT CGTCGCCCCA TTTATCCCTG   691
AN8530 cDNA sequenced   CACAAGCATT GCGCATGTGG CCAACCTTCA TGCGACGCCT CGTCGCCCCA TTTATCCCTG   691

AN8530 Genomic          GTGTCCAGAA GATCCGCGCG GAGCTCGAGG AGGCCCGTCG TATCATTCTC CCTGTATTGG   900
AN8530 cDNA             GTGTCCAGAA GATCCGCGCG GAGCTCGAGG AGGCCCGTCG TATCATTCTC CCTGTATTGG   751
AN8530 cDNA sequenced   GTGTCCAGAA GATCCGCGCG GAGCTCGAGG AGGCCCGTCG TATCATTCTC CCTGTATTGG   751

AN8530 Genomic          AAAAGCGCAA GGCAGAAAAA CAAACTGCTA TTGCGGCTGG CAAGACCCCA GCTCGGTACA   960
AN8530 cDNA             AAAAGCGCAA GGCAGAAAAA CAAACTGCTA TTGCGGCTGG CAAGACCCCA GCTCGGTACA   811
AN8530 cDNA sequenced   AAAAGCGCAA GGCAGAAAAA CAAACTGCTA TTGCGGCTGG CAAGACCCCA GCTCGGTACA   811

AN8530 Genomic          ACGACGCGAT GGAATGGATG GAACAGTGTG CTAAGGGGCG TCCGTATGAT GCGGCGGTGT   1020
AN8530 cDNA             ACGACGCGAT GGAATGGATG GAACAGTGTG CTAAGGGGCG TCCGTATGAT GCGGCGGTGT   871
AN8530 cDNA sequenced   ACGACGCGAT GGAATGGATG GAACAGTGTG CTAAGGGGCG TCCGTATGAT GCGGCGGTGT   871

AN8530 Genomic          CGCAGCTTTC TCTCTCACTG GGCGCTATCC ATACCACATC CGACATGCTG ACCCAGGTTC   1080
```

```
AN8530 cDNA            CGCAGCTTTC TCTCTCACTG GGCGCTATCC ATACCACATC CGACATGCTG ACCCAGGTTC  931
AN8530 cDNA sequenced  CGCAGCTTTC TCTCTCACTG GGCGCTATCC ATACCACATC CGACATGCTG ACCCAGGTTC  931

AN8530 Genomic         TCTACGACAT TTGCGGACAC AGCGATCTGG TCGATGAGCT CAGGCAGGAG GTTCTCACCG  1140
AN8530 cDNA            TCTACGACAT TTGCGGACAC AGCGATCTGG TCGATGAGCT CAGGCAGGAG GTTCTCACCG  991
AN8530 cDNA sequenced  TCTACGACAT TTGCGGACAC AGCGATCTGG TCGATGAGCT CAGGCAGGAG GTTCTCACCG  991

AN8530 Genomic         TCATTGCGGC GGAAGGATGG CAGAAGACGA CTCTGTACAA ACTGAAGTTG ATGGATAGCG  1200
AN8530 cDNA            TCATTGCGGC GGAAGGATGG CAGAAGACGA CTCTGTACAA ACTGAAGTTG ATGGATAGCG  1051
AN8530 cDNA sequenced  TCATTGCGGC GGAAGGATGG CAGAAGACGA CTCTGTACAA ACTGAAGTTG ATGGATAGCG  1051

AN8530 Genomic         TTCTCAAGGA AAGCCAAAGG GTAAAACCCA TCGGGATCGG TACGTACGCT CCTCACACAG  1260
AN8530 cDNA            TTCTCAAGGA AAGCCAAAGG GTAAAACCCA TCGGGATCG- ---------- ----------  1090
AN8530 cDNA sequenced  TTCTCAAGGA AAGCCAAAGG GTAAAACCCA TCGGGATCG- ---------- ----------  1090

AN8530 Genomic         GTACAACGTC TGCTAACGAG TCAGCAACGA TGCACCGCTT GGCTGAAAAA ACAGTCAAGC  1320
AN8530 cDNA            ---------- ---------- ---------- ---------- ---------- ----TCAAGC  1096
AN8530 cDNA sequenced  ---------- ---------- ----CAACGA TGCACCGCTT GGCTGAAAAA ACAGTCAAGC  1126

AN8530 Genomic         TTTCTGACGG CACTGTTATC CCCAAAAACG CCAACCTGAT CGTTTCCTCG CAGCGGATGT  1380
AN8530 cDNA            TTTCTGACGG CACTGTTATC CCCAAAAACG CCAACCTGAT CGTTTCCTCG CAGCGGATGT  1156
AN8530 cDNA sequenced  TTTCTGACGG CACTGTTATC CCCAAAAACG CCAACCTGAT CGTTTCCTCG CAGCGGATGT  1186

AN8530 Genomic         GGGATGAGAG CATCTACCCC TCCCCAGACA AATTCGACCC TTACCGCTTC CTTAGATTGC  1440
AN8530 cDNA            GGGATGAGAG CATCTACCCC TCCCCAGACA AATTCGACCC TTACCGCTTC CTTAGATTGC  1216
AN8530 cDNA sequenced  GGGATGAGAG CATCTACCCC TCCCCAGACA AATTCGACCC TTACCGCTTC CTTAGATTGC  1246

AN8530 Genomic         GAGAGACGCC TGGTCATGAG ACTTCGGCCC AGTTCGTCTC ACCGTCGCCA GATCATATGG  1500
AN8530 cDNA            GAGAGACGCC TGGTCATGAG ACTTCGGCCC AGTTCGTCTC ACCGTCGCCA GATCATATGG  1276
AN8530 cDNA sequenced  GAGAGACGCC TGGTCATGAG ACTTCGGCCC AGTTCGTCTC ACCGTCGCCA GATCATATGG  1306

AN8530 Genomic         GCTTTGGGTT TGGCAAACAC TCCTGTCCTG GCCGCTTCTT CGCTGCGAAC GAGATCAAGA  1560
AN8530 cDNA            GCTTTGGGTT TGGCAAACAC TCCTGTCCTG GCCGCTTCTT CGCTGCGAAC GAGATCAAGA  1336
AN8530 cDNA sequenced  GCTTTGGGTT TGGCAAACAC TCCTGTCCTG GCCGCTTCTT CGCTGCGAAC GAGATCAAGA  1366

AN8530 Genomic         TCGCGCTCTG CCATATTCTG CTCAAGTATG ACTTCCGACT GACGGAAGAA TGGAGAAATC  1620
AN8530 cDNA            TCGCGCTCTG CCATATTCTG CTCAAGTATG ACTTCCGACT GACGGAAGAA TGGAGAAATC  1396
AN8530 cDNA sequenced  TCGCGCTCTG CCATATTCTG CTCAAGTATG ACTTCCGACT GACGGAAGAA TGGAGAAATC  1426

AN8530 Genomic         CGCGGCCGAT TGCAAGTGGT GCAGGGTTGA CTGCTGAGCC CAGAGCGACG ATGGAGATTC  1680
AN8530 cDNA            CGCGGCCGAT TGCAAGTGGT GCAGGGTTGA CTGCTGAGCC CAGAGCGACG ATGGAGATTC  1456
AN8530 cDNA sequenced  CGCGGCCGAT TGCAAGTGGT GCAGGGTTGA CTGCTGAGCC CAGAGCGACG ATGGAGATTC  1486

AN8530 Genomic         GCAGGAGAAA AGAGGAAATT CAGTTATAG 1709
AN8530 cDNA            GCAGGAGAAA AGAGGAAATT CAGTTATAG 1485
AN8530 cDNA sequenced  GCAGGAGAAA AGAGGAAATT CAGTTATAG 1515
```
